# The relationship between foot and ankle joint flexibility measures and barefoot plantar pressures in healthy older adults: a cross-sectional study

**DOI:** 10.1186/s12891-022-05618-w

**Published:** 2022-07-30

**Authors:** Bonnie McNab, Sean Sadler, Sean Lanting, Vivienne Chuter

**Affiliations:** 1 School of Health Sciences, Faculty of Health and Medicine, University of Newcastle, Central Coast Campus, 10 Chittaway Road, Ourimbah, Callaghan, NSW 2258 Australia; 2 Discipline of Podiatry, School of Health Science, Western Sydney University, Campbelltown, NSW Australia

**Keywords:** Ankle, Equinus, Foot, Plantar pressure, Older adults

## Abstract

**Background:**

Restriction in foot and ankle joint range of motion, such as an ankle equinus, has been associated with increased plantar pressure and its complications. However, previous research is limited by its scope of measures and study populations. The aim of this study was to investigate the relationship between foot and ankle joint range of motion on barefoot plantar pressures during walking in healthy older adults.

**Methods:**

This cross-sectional study recruited 49 older adults. Participants underwent measures of foot (first metatarsophalangeal dorsiflexion range of motion, and navicular drop and drift) and ankle joint range of motion, foot posture, body mass index, and plantar pressure during barefoot walking. Spearman Rank Order Correlations were used to explore the relationship between foot and ankle measures, body mass index, and plantar pressure, with significant correlations explored in a hierarchical regression analysis. A Mann-Whitney *U* test was performed to compare plantar pressure values between those with and without ankle equinus per region of the foot.

**Results:**

Mean (SD) age and BMI were 72.4 years (5.2) and 29.8 kg/m^2^ (5.9) respectively. A total of 32 of the 49 participants (65%) identified as female sex. Mean (SD) ankle joint range of motion was 32.7 (6.4) degrees with 17/49 (34.7%) participants classified as having an ankle equinus (defined as < 30 degrees of ankle joint dorsiflexion range of motion). We found that an ankle equinus predicted a statistically significant amount of peak forefoot plantar pressure (*p* = 0.03). Participants with an ankle equinus displayed significantly higher forefoot peak pressure 677.8 kPa (589.9 to 810.4) compared to those with no equinus 565.58 kPa (447.3 to 651.2), *p* = 0.02. A statistically significant correlation was found between body mass index and midfoot peak pressure (*p* < 0.01) and pressure-time integral (*p* < 0.01). No other significant correlations were found.

**Conclusion:**

Clinicians should consider screening for an ankle equinus and body mass index as a simple way to identify which healthy older adults may be at risk of pressure-related complications in the mid- and forefoot.

## Introduction

Within the ageing population, foot and ankle restriction has been linked to specific patterns of increases in plantar pressure. For example, restriction in ankle joint dorsiflexion range of motion, referred to as an ankle equinus, has been associated with increased forefoot plantar pressures [1–3]. Elevated forefoot pressures are likely due to contracture of the Achilles tendon, which in turn places increased loading on the forefoot during dynamic gait [4, 5]. Similarly, a reduction in motion in the first metatarsophalangeal joint, which is associated with a loss of high-gear propulsion, has been linked to increased plantar pressures within the hallux region [6]. Increased plantar pressures within these regions of the foot have been linked to pressure-related complications such as foot pain and ulceration [1, 2, 7, 8].

At the midfoot, static measures of navicular drop have been associated with hallux and medial forefoot plantar pressures, whilst navicular drift has been associated with hallux pressure only [9]. In addition, people with a supinated (high arched) foot type have been found to have higher plantar pressure on the lateral forefoot, while those with pronated feet had higher plantar pressure in the midfoot and medial forefoot regions [10]. However, of the available literature, the focus has been on individual foot and ankle measures or different techniques, such as a flexed knee lunge for ankle joint range of motion in older adults [11], in heterogeneous populations with some including a mix of people with and without diabetes [2, 11], or other conditions known to affect plantar pressures such as sensory loss [12]. Additionally, these studies have not considered the influence of body mass on plantar pressure. This may be significant as previous research has found that obesity is associated with increased peak pressure [13]. Based on these previous studies, the effect of limited ankle and foot range of motion measures on barefoot plantar pressure during walking gait in healthy older adults is unclear. Understanding the relationship between these foot and ankle measures and plantar pressures in healthy older adults may lead to a better understanding of how both foot type and restriction within these joints may contribute to elevated plantar pressures. This may help clinicians in the assessment and management of this population.

Therefore, the primary aim of this study was to investigate the relationship between weight bearing ankle joint dorsiflexion range of motion (equinus vs no equinus) and barefoot plantar pressures during walking in healthy adults aged 65 years and over. Secondary aims were to explore the association between foot type, navicular drop and drift, first metatarsophalangeal joint dorsiflexion range of motion, body mass index (BMI), and barefoot plantar pressures during walking within the same population.

## Methods

The University of Newcastle Human Research Committee granted ethics approval (H-2015-0354) and written informed consent was obtained from all participants prior to their inclusion. Participants were recruited by convenience sampling from the Central Coast community or the University of Newcastle Student Podiatry Clinic at Wyong Hospital, New South Wales, Australia on a volunteer basis, from February 2019 to March 2020. Data were collected during a one-off testing session at either the University of Newcastle Student Podiatry Clinic at Wyong Hospital or the University of Newcastle Ourimbah campus. Older adults, aged 65 years and over, who were able to read and speak basic English were eligible for inclusion. People with endocrine (e.g. diabetes mellitus), autoimmune (e.g. rheumatoid arthritis), vascular (e.g. peripheral arterial disease, ulceration, stroke), or neurological (Charcot-Marie-Tooth) conditions were excluded due to the potential for each of these conditions to affect plantar pressure. Additionally, any participant with known lower leg or foot sensory loss (e.g. from chemotherapy or alcoholic-related neuropathy), a history of surgery in the lower limb (e.g. arthrodesis), significant trauma or amputation of the foot or ankle was ineligible for the same reason. Potential participants who were unable to ambulate 10 m unaided were ineligible due to study protocol requirements.

### Procedures

Participants self-reported and recorded their medical and demographic information on a custom-made questionnaire developed by the authors for this study. The International Physical Activity Questionnaire (IPAQ-7) was used to measure levels of physical activity [14]. The IPAQ-7 examines activity levels over the last 7 days including the number of days and time spent being physically active and categorises participants as low, moderate, or highly active based on the total volume and number of days of each level of physical activity.

Weight and height were measured using basic bathroom scales and a wall-mounted tape measure, respectively. All lower limb measurements were performed on the participant’s dominant side to adhere to the assumption of independence of data [15]. Ankle dorsiflexion range of motion was measured in degrees using the weight-bearing lunge test with the knee extended and a digital inclinometer (Bear Digital Protractor 82201B-00, China) placed on the anterior border of the tibia, 15 cm distal to the tibial tuberosity [16]. To standardise testing, the participant was required to keep both feet perpendicular to the wall and maintain full heel contact with the ground. Three measurements were taken with the average of the three used for data analysis. First metatarsophalangeal joint dorsiflexion range of motion was measured non-weight bearing using a hand held tractograph [2, 17, 18]. This assessment was completed three times with the average of the three used for data analysis. Navicular drop and drift were measured once to assess the sagittal and transverse plane displacement of the midfoot and rearfoot [19]. Participants stood on a sheet of A4 paper, and had the medial aspect of the navicular tuberosity marked. Once in subtalar joint neutral (NCSP), the height of the navicular tuberosity from the ground was measured and then again in a relaxed calcaneal stance position (RCSP), with the difference representing navicular drop [19]. Similarly, the transverse displacement, as measured by the horizontal distance marked on the paper between NCSP and RCSP, represented navicular drift. A detailed description of the method used for Foot Posture Index (FPI) has been previously reported [20]. Briefly, the FPI was used to determine foot type and consists of six criteria, which are graded on a 5 point scale from − 2 to 2, with the score for each criterion summed to create a total score [21].

Barefoot plantar pressures were collected with the Tekscan HR MatTM (Tekscan Inc., South Boston, USA) with the mat placed on top of a firm flat floor. The mat was calibrated for each participant using their bodyweight prior to testing and the sampling rate was 60 Hz. The two-step protocol was used for data collection [22–24]. The mat was positioned so that participants were able to commence walking by stepping forward with their non-dominant side and their second step would result in their dominant side striking the mat. Participants then continued to walk another 3 or 4 steps to a tape mark on the floor four metres from the start position. Participants were allowed to practise before data collection to familiarise themselves with the procedure and to position the mat correctly. To avoid targeting of the mat, participants were asked to look straight ahead and to walk at their normal walking speed during the barefoot pressure assessment. Four successful trials were recorded for data analysis [23]. Walking speed was measured with a digital stopwatch from the point of step-off to the point where both feet passed the mark 4 m from the start position. If inter-trial speed differed by more than 5%, it was rejected. To evaluate barefoot plantar pressure, the foot was divided into masks or five distinct regions: hallux, digits 2–5, forefoot, midfoot and rearfoot. This method of masking is similar to that of a previous study [25] however, we consolidated the three metatarsophalangeal joints (M1, M2, M3–5) into the one forefoot region, as has been described previously [3].

### Outcome measures

The primary outcome was the effect of ankle joint dorsiflexion range of motion (equinus vs no equinus) on barefoot peak plantar pressure and pressure-time integral under the hallux, forefoot, midfoot and rearfoot. Plantar pressure data were not included for the second through to fifth digits due to difficulty in accurately capturing these data [16]. The secondary outcome was the association between first metatarsophalangeal joint dorsiflexion range of motion, navicular drop and drift, FPI, BMI and peak pressure and pressure time-integral in the same regions of the foot.

### Statistical analyses

Plantar pressure data were analysed in Tekscan software (Tekscan Inc., South Boston, USA) and then entered into Microsoft Excel along with other participant data. Data were then exported to the statistical package for the social sciences (version 25.0 Chicago, Illinois, USA) for analysis. Data were assessed for normality using histograms, boxplots, and the Shapiro-Wilk test. Means and standard deviations were reported for demographic, anthropometric, range of motion and foot type data. Plantar pressure data were non-normally distributed, so medians and interquartile ranges were used.

Spearman Rank Order Correlation was used to investigate the relationship between ankle joint dorsiflexion range of motion and peak pressure and pressure time-integral in each region of the foot. Ankle joint range of motion was dichotomised into equinus vs no equinus, with < 30 degrees indicating the presence of equinus [16, 26]. For the primary outcome, statistically significant correlations (*p <* 0.05) were further explored in a hierarchical regression analysis, with age, sex, and BMI used in the first step to account for any potential confounding effect. Additionally, and after controlling for these variables, the ability of an ankle equinus to predict differences in plantar pressure between those with and without an ankle equinus was explored using the regression coefficients and their 95% confidence intervals. A Mann-Whitney U test was performed to compare plantar pressure values between those with and without ankle equinus per region of the foot. Effect sizes for differences in plantar pressure variables were calculated using Microsoft Excel and reported using the z value statistic divided by the square root of N (total cases) with the size of the effect (r) interpreted according to Cohen: 0.1–0.29 = small effect; 0.3–0.49 = medium effect; ≥0.5 = large effect [27].

For the secondary outcomes, Spearman Rank Order Correlation was used to investigate the association between first metatarsophalangeal dorsiflexion joint range of motion, navicular drop and drift, foot type, BMI and plantar pressures. FPI values were converted to Rasch foot type values so that these data could be treated as continuous [28]. The strength of the correlation was interpreted as small (*r =* 0.10–0.29), moderate (*r =* 0.30–0.49), and large (*r =* 0.50–1.0) [27]. All assumptions for statistical analyses were met.

## Results

Forty-nine adults aged between 67 and 78 with no conditions known to affect plantar pressures were recruited for the study (Table 1). Most participants were classified as overweight according to their BMI, and self-reported moderate to high physical activity levels.

**Table 1:** Participant characteristics. All values are mean (SD) unless otherwise specified

Age, years	72.4 (5.2)
Female, n (% of total)	32 (65.0)
BMI, kg/m^2^	29.8 (5.9)
Physical activity level (IPAQ-7)	
Low, n (% of total)	14 (28.5)
Moderate, n (% of total)	17 (34.7)
High, n (% of total)	18 (36.7)
Ankle joint range of motion in deg, mean (SD), [range]	32.7 (6.4), [19.0 to 48.3]
Ankle Equinus < 30 deg, n (% of total)	17 (34.7%)
Ankle joint range of motion in deg, [range]	26.1 (3.8), [19.0 to 29.9]
Foot type (Rasch values), *n =* 26	
Cavus FPI < -0.21, n (% of total), mean (range)	1 (3.8%), −1.54 (−1.54–1.54)
Neutral FPI −0.21 to 2.98, n (% of total), mean (range)	13 (50.0%), 1.79 (0.50 to 2.98)
Planus FPI > 2.98, n (% of total), mean (range)	12 (46.0%), 5.22 (3.81 to 7.77)
Mean (range)	3.2 (2.3)
Navicular Drop, mm, *n =* 26	8.2 (5.4)
Navicular Drift, mm, *n =* 26	8.6 (4.2)
1st MPJ dorsiflexion ROM, deg, *n =* 26	78.5 (25.2)

*SD* Standard deviation, *BMI* Body mass index, *IPAQ-7* Short version of the international physical activity questionnaire, *FPI* Foot posture index, *MPJ* Metatarsophalangeal joint, *ROM* Range of motion.

Ankle equinus (< 30 degrees dorsiflexion range of motion) had a significant, moderate correlation with higher forefoot peak pressure (*r =* 0.327, *n =* 49, *p =* 0.02) (Table 2). Correlations between ankle equinus and other regions of the foot, for both peak pressure and pressure-time integral, were not significant (Table 2).

**Table 2:** Spearman correlation between ankle equinus and peak plantar pressure and pressure-time integral (*n =* 49)

	Rearfoot	Midfoot	Forefoot	Hallux
Ankle equinus (peak pressure)	0.041	0.138	0.327*	0.121
Ankle equinus (pressure-time integral)	0.167	0.136	0.194	−0.015

*Correlation is significant at the 0.05 level (2-tailed)

A hierarchical regression analysis was used to assess the relative contribution that an ankle equinus makes to forefoot peak plantar pressure after controlling for age, sex, and BMI. In step 1, these control variables explained 4.7% of the variance in forefoot peak plantar pressure values. In step 2, ankle equinus was entered and the total variance explained by the model as a whole was 12.3%, F (4, 44) = 2.69, *p =* 0.04. After controlling for age, sex, and BMI, ankle equinus explained a further 9.0% of the variance in peak forefoot plantar pressure, R square change = 0.09, F change (1, 44) = 4.94, *p =* 0.03. Additionally, based on the regression analysis and after controlling for confounders, participants with an ankle equinus were predicted to have more peak pressure in their forefoot (153.28 kPa [95%CI 14.23 to 292.33]) compared to those without an ankle equinus (*p =* 0.03).

Participants with an ankle equinus were found to have a significantly higher peak forefoot plantar pressure (677.8 kPa [589.9 to 810.4]) compared to those without an ankle equinus (565.5 kPa [447.3 to 651.2]), with a medium effect size (effect size *r =* 0.32) (Table 3). Plantar pressure values in Table 3 are not adjusted for age, sex, and BMI due to the number of participants in each group.

**Table 3:** Unadjusted plantar pressure variables between groups. All values are median (IQR) unless otherwise specified

	All participants, *n =* 49	Equinus, *n =* 17	No Equinus, *n =* 32	*Z score*	*p* value
Peak pressure, kPa					
Rearfoot	437.8 (346.0 to 505.8)	437.4 (347.2 to 489.0)	437.8 (345.5 to 564.0)	−0.28	0.78
Midfoot	93.8 (58.8 to 135.0)	93.4 (54.8 to 131.9)	115.3 (61.6 to 176.4)	−0.96	0.34
Forefoot	609.8 (466.0 to 729.5)	677.8 (589.9 to 810.4)	565.5 (447.3 to 651.2)	−2.27	**0.02**
Hallux	494.0 (330.0 to 579.0)	496.0 (282.6 to 563.4)	494.0 (347.9 to 617.8)	−0.84	0.40
Pressure-time integral, kPa/s					
Rearfoot	65.5 (53.0 to 81.8)	61.7 (50.5 to 79.8)	66.5 (56.4 to 87.2)	−1.16	0.25
Midfoot	17.3 (10.0 to 25.8)	16.7 (7.9 to 25.3)	19.5 (12.9 to 27.9)	−0.95	0.34
Forefoot	63.0 (55.7 to 80.8)	60.9 (54.8 to 73.8)	70.9 (56.5 to 95.7)	−1.34	0.18
Hallux	41.7 (28.9 to 58.3)	43.7 (25.6 to 63.6)	37.2 (30.5 to 50.2)	−0.11	0.92

*IQR* Inter quartile range, *kPa* Kilopascals, *kPa/s* Kilopascals per second.

Correlations for the secondary outcomes are presented in Table 4. A statistically significant, moderate correlation was found between BMI and midfoot peak pressure (*r =* 0.499, *n =* 49, *p <* 0.01). A statistically significant, large correlation was found between BMI and midfoot pressure-time integral (*r =* 0.558, *n =* 49, *p <* 0.01).

**Table 4:** Spearman correlation between foot measures and plantar pressure values

	Rasch foot posture index, *n =* 26	Navicular drop, *n =* 26	Navicular drift, *n =* 26	1st MPJ, *n =* 26	Body Mass Index (BMI), *n =* 49
Peak pressure, kPa					
Rearfoot	0.076	−0.087	− 0.113	− 0.087	−0.112
Midfoot	0.189	0.021	0.060	−0.327	0.499**
Forefoot	0.161	−0.068	0.041	−0.102	−0.085
Hallux	0.371	0.383	0.244	−0.013	−0.135
Pressure-time integral, kPa/s					
Rearfoot	0.379	0.115	0.089	0.066	0.272
Midfoot	0.337	0.198	0.292	−0.199	0.558**
Forefoot	0.204	−0.004	0.004	0.065	0.019
Hallux	0.308	0.239	−0.053	− 0.150	−0.026

** Correlation is significant at the 0.01 level (2-tailed)

*BMI* Body mass index, *kPa* Kilopascals, *kPa/s* Kilopascals per second.

## Discussion

This study primarily explored the relationship between a weight bearing ankle equinus and barefoot plantar pressures in adults aged 65 years and over and investigated differences in plantar pressure values in these participants with and without an ankle equinus. We found that after controlling for age, sex, and BMI, the regression model predicted that those with a weight bearing ankle equinus had more peak pressure in their forefoot (153.28 kPa [95%CI 14.23 to 292.33]) compared to those without an ankle equinus (*p =* 0.03). We also found that participants with an ankle equinus had significantly higher forefoot peak pressure during barefoot walking compared to those without an ankle equinus. Ankle equinus was not able to significantly predict peak pressure or pressure-time integral values in any other region of the foot. Additionally, there were no significant differences in peak pressure or pressure-time integral values in any other region of the foot for those with or without a weight bearing ankle equinus.

Comparison of our findings with the limited existing research is difficult due to differences in participant populations, techniques used to measure ankle joint range of motion, and the inconsistent thresholds to determine an ankle equinus. In contrast to our findings, a previous trial did not find the same relationship between ankle joint range of motion and forefoot peak plantar pressure [11]. This may be due to the previous study measuring ankle joint range of motion with the knee flexed. We measured ankle joint dorsiflexion range of motion with the knee extended to include gastrocnemius tightness, which is thought to be the most prevalent cause of ankle dorsiflexion restriction [5]. To aid clinical interpretation, we classified participants as having an ankle equinus (< 30 degrees of dorsiflexion) or no equinus. This dichotomisation of participants may have resulted in two groups that were more likely to be different. This is supported by our findings of significantly higher peak forefoot plantar pressure values in those with ankle equinus, compared to those without ankle equinus. Our results suggest that in older adults, clinicians should consider assessment of weight bearing ankle joint dorsiflexion range of motion, with the knee extended, as a way to determine if individuals may be experiencing elevated forefoot peak plantar pressure, particularly where there may be relevant symptoms and especially if the range of motion is < 30 degrees.

We also investigated the association between first metatarsophalangeal joint range of motion, navicular drop and drift, foot type, and barefoot plantar pressures. There was no significant correlations found between these measures and barefoot peak pressure or pressure-time integral. In contrast, a previous study found significant correlations between reduced first metatarsophalangeal joint range of motion and higher barefoot peak pressure under the medial forefoot, as well as between a pes planus foot type and higher peak pressure in the midfoot [11]. Previous research investigating navicular drop and drift has demonstrated similar findings that indicate that those with flatter feet (as determined by increased navicular drop and drift) experience significantly higher medial column peak pressure [9]. However, we did not find the same relationship between navicular drop and drift, foot type, and peak pressure or pressure-time integral. A possible reason for these different findings is likely due to the smaller sample size in our study compared to the previous research [9, 11], as well as previous research including younger adults [9]. In addition, an older population is likely to experience higher rates of forefoot deformity due to development of structural changes including hallux valgus and more generalised osteoarthritic changes [1]. While the mean range of motion at the first metatarsophalangeal joint for this study was within normal limits, suggesting most did not have osteoarthritic restrictions at this joint, presence of deformity was not captured as part of this study. Such changes are likely to alter loading patterns underneath the forefoot and may explain the difference seen in previous research including younger adults.

We found a significant, moderate to large correlation between BMI and midfoot peak pressure, and BMI and midfoot pressure-time integral. This finding is in agreement with previous research that found older obese women had higher peak pressure in the midfoot compared to older non-obese women [13]. This previous study measured body mass using dual-energy x-ray absorptiometry compared to our study, which calculated BMI based on clinical measures of height and weight (Kg/m^2^). These findings may have clinical implications in the development of foot pain particularly through the midfoot in people who are overweight or obese. Furthermore, within diabetic populations the effect of obesity on plantar pressures has not yet been conclusively established. Significant contribution of increasing levels of obesity to plantar pressures has high relevance to prevention of pressure-related diabetic foot complications.

In clinical practice, stretching of the gastrocnemius and soleus muscles are commonly prescribed treatments to help reduce calf muscle tightness and increase ankle joint dorsiflexion range of motion. Previous studies have found that calf muscle stretching is effective at increasing ankle joint dorsiflexion range of motion [29, 30]. However, it is yet to be determined whether these changes in ankle joint range of motion lead to lower forefoot peak pressure or pressure time-integral. Therefore, until further research is performed, clinicians should also consider other conservative therapies that have been shown to help reduce forefoot plantar pressures, such as wearing sport shoes [31], in-shoe pads [32], and shoe insoles [33].

The results of this study should be considered in the context of several limitations. Peak pressure and pressure time-integral data were measured barefoot, with no in-shoe measurement performed. Whilst this provides important information on the pressures associated with barefoot walking, it may not be reflective of plantar pressures that healthy older adults would typically experience when ambulating in their own footwear. This, coupled with the healthy older adult population with a BMI indicating overweight/obesity and self-reported moderate to high levels of physical activity, limits the generalisability of this study’s findings. Additionally, we only controlled for inter-trial walking speed for the same participant, not between participants, however this was considered to reflect the everyday walking conditions of the participants more accurately and is consistent with other research [34]. Measures used in this project including ankle equinus have previously been shown to be reliable [17–19, 23, 25, 35, 36], but it should be recognised that operator error may have affected the results. Data on the amount of time spent barefoot by participants, along with digital deformity and foot muscle strength were not collected. These variables may provide further information on plantar pressure distribution in our study population but were not the aims of this study. Finally, consideration of the study’s sample size is needed, specifically for the secondary foot-related measures of this study.

## Conclusion

We found that a weight bearing ankle equinus (< 30° dorsiflexion range of motion) significantly predicted forefoot peak plantar pressure during barefoot walking, and resulted in higher forefoot peak pressure values compared to those without an ankle equinus. BMI was significantly correlated with midfoot peak pressure and pressure-time integral, but the foot type and foot joint range of motion measures explored in this study were not significantly associated with plantar pressure values. Screening for an ankle equinus and BMI may be a simple way to identify healthy older adults at risk for pressure-related complications in the mid- and forefoot. Future research should consider the effect of digital deformities and foot muscle strength on plantar pressure. Additionally, future research should aim to investigate the effectiveness of a calf-specific stretching intervention on reducing forefoot peak pressure and pressure time-integral in both shod and unshod healthy older adult populations.

## Data Availability

The datasets used and/or analysed during the current study are available from the corresponding author on reasonable request.

## Abbreviations

BMI: Body mass index
FPI: Foot posture index
IPAQ-7: Short version of the international physical activity questionnaire
IQR: Interquartile range
kPa: Kilopascals
kPa/s: Kilopascals per second
MPJ: Metatarsophalangeal joint
NCSP: Neutral calcaneal stance position
ROM: Range of motion
RSCP: Resting calcaneal stance position
SD: Standard deviation
SPSS: Statistical package for the social sciences
95%CI: 95% confidence interval
